# Recent advances in understanding amyotrophic lateral sclerosis and emerging therapies

**DOI:** 10.12703/b/9-12

**Published:** 2020-11-17

**Authors:** Lauren M Gittings, Rita Sattler

**Affiliations:** 1 Department of Neurobiology, Barrow Neurological Institute, Phoenix, AZ, USA

**Keywords:** ALS, Amyotrophic lateral sclerosis, ALS therapeutics, clinical trials, gene therapy, immunotherapy, platform trials

## Abstract

Amyotrophic lateral sclerosis (ALS) is a fatal neurodegenerative disease that is characterized by degeneration of both upper and lower motor neurons and subsequent progressive loss of muscle function. Within the last decade, significant progress has been made in the understanding of the etiology and pathobiology of the disease; however, treatment options remain limited and only two drugs, which exert a modest effect on survival, are approved for ALS treatment in the US. Therefore, the search for effective ALS therapies continues, and over 60 clinical trials are in progress for patients with ALS and other therapeutics are at the pre-clinical stage of development. Recent advances in understanding the genetics, pathology, and molecular mechanisms of ALS have led to the identification of novel targets and strategies that are being used in emerging ALS therapeutic interventions. Here, we review the current status and mechanisms of action of a selection of emerging ALS therapies in pre-clinical or early clinical development, including gene therapy, immunotherapy, and strategies that target neuroinflammation, phase separation, and protein clearance.

## Introduction

Amyotrophic lateral sclerosis (ALS) is a fatal neurodegenerative disease characterized by progressive loss of upper motor neurons in the motor cortex and lower motor neurons in the brainstem and anterior horn of the spinal cord. Clinically, this manifests as weakness and atrophy of voluntary muscles, resulting in the loss of the ability to walk, speak, and swallow as the disease progresses^1^. Additionally, it is estimated that 20 to 50% of patients with ALS present with cognitive impairments that would meet the diagnostic criteria for probable or definite frontotemporal dementia (FTD)^2^. Disease prognosis is poor; most patients with ALS die within 3 to 5 years after the diagnosis; this is typically due to respiratory failure resulting from the progressive weakening of respiratory muscles^1^. Pathologically, more than 97% of ALS cases are characterized by inclusions of the nuclear RNA-binding protein (RBP) TAR DNA-binding protein 43 (TDP-43) in the cytoplasm of neurons and glia. The few cases that do not exhibit TDP-43 pathology present with pathological inclusions of either the superoxide dismutase (SOD1) or fused in sarcoma (FUS) protein^3^.

ALS can be broadly separated into two categories—familial ALS (fALS) (10–15%) and sporadic ALS (sALS) (85–90%)—depending on whether there is a family history of ALS^4^. At least 50 potential causative or disease-modifying genes have been linked to ALS^5^, but a G_4_C_2_ hexanucleotide repeat expansion in the *C9orf72* gene is the most common ALS-causing mutation identified to date, accounting for about 40% of fALS cases and 6 to 8% of sALS cases in Caucasian populations^6^. Other commonly mutated genes in ALS include *SOD1*, *TARDPB*, or *FUS*, and variants in other disease-causing ALS-linked genes are relatively uncommon^4,7^. ALS linked to mutations in these genes is hypothesized to be caused by loss-of-function or toxic gain-of-function of the protein products of these genes and subsequent downstream cellular consequences^8^. In contrast, three non-mutually exclusive mechanisms have been proposed as a potential cause of *C9orf72*-linked ALS: loss-of-function of the *C9orf72* protein, sequestration of essential RBPs by foci containing G_4_C_2_-containing RNA, and toxicity induced by one or more dipeptide repeat (DPR) proteins^9,10^. These DPR proteins are produced as a result of bidirectional repeat-associated non-AUG initiated (RAN) translation of the G_4_C_2_ hexanucleotide repeat expansion and accumulate in p62-positive, TDP-43-negative pathological aggregates in *C9orf72* repeat expansion carriers^11–13^.

Although research into genetic forms of ALS has implicated several dysregulated biological pathways in ALS pathogenesis, the precise mechanism of disease is unknown. The lack of a defined cause of disease is reflected in the number of currently available therapeutics for ALS. Despite many previous and ongoing clinical trials of various drugs targeting different biological mechanisms (comprehensively reviewed in 14 and 15), only two approved treatments are currently in widespread use for ALS: the anti-excitotoxic drug riluzole and edaravone, whose mechanism of action is unknown but presumed to be through its antioxidant properties^16,17^.

Here, we present a collection of emerging therapeutic approaches based on a selection of recent novel pre-clinical discoveries covering a broad range of ALS disease mechanisms or genetics (or both). A summary of the therapeutic approaches that are discussed in this review and have entered clinical trials is provided in Table 1.

**Table 1.** Summary of therapeutic approaches that have entered clinical trials for the treatment of amyotrophic lateral sclerosis.

Therapeutic	Therapeutic approach	Therapeutic target	Class of drug	Eligible ALS population	Trial phase	ClinicalTrials.gov Identifier
Tofersen (BIIB067)	Gene therapy: antisense oligonucleotide	SOD1 transcript	Antisense oligonucleotide	SOD1 ALS	III	NCT02623699
BIIB078	Gene therapy: antisense oligonucleotide	C9orf72 repeat expansion	Antisense oligonucleotide	C9orf72 ALS	I	NCT03626012
Masitinib	Modulating neuroinflammation	Tyrosine kinase inhibitor	Small molecule	Familial or sporadic ALS	III	NCT03127267
Ibudilast (MN-166)	Modulating neuroinflammation	Phosphodiesterase inhibitor	Small molecule	Familial or sporadic ALS	II / III	NCT04057898
Fasudil	Modulating neuroinflammation	Rho kinase inhibitor	Small molecule	Not specified	II	NCT03792490
Ravulizumab	Modulating neuroinflammation	Complement component 5 inhibitor	Monoclonal antibody	Familial or sporadic ALS	III	NCT04248465
Zilucoplan	Modulating neuroinflammation	Complement component 5 inhibitor	Small molecule	Familial or sporadic ALS	II / III	NCT04297683
Anakinra	Modulating neuroinflammation	IL-1 receptor antagonist	Monoclonal antibody	Familial or sporadic ALS	II	NCT01277315
Tocilizumab	Modulating neuroinflammation	IL-1 receptor antagonist	Monoclonal antibody	Not specified	II	NCT02469896
Arimoclomol	Clearance of protein aggregates	Upregulates heat shock proteins	Small molecule	Not specified	III	NCT03491462
Colchicine	Clearance of protein aggregates	Enhances expression of HSPB8	Small molecule	Sporadic ALS	II	NCT03693781
Rapamycin	Clearance of protein aggregates	Stimulate autophagy	Small molecule	Familial or sporadic ALS	II	NCT03359538
BIIB100/KPT- 350	Targeting nucleocytoplasmic transport	XPO1/CRM1 inhibitor	Small molecule	Not specified	I	NCT03945279
HEALEY platform trial	Various	Various	Various	Familial or sporadic ALS	II / III	NCT04297683

ALS, amyotrophic lateral sclerosis; IL-1, interleukin 1; SOD1, superoxide dismutase; XPO1, exportin 1.

## Gene therapies

The heritability of fALS makes the disease a promising candidate for gene therapy; as such, clinical trials that use the principles of gene therapy are under way in fALS patients carrying *SOD1* (NCT02623699) and *C9orf72* (NCT03626012) mutations. Gene therapy is a broad term that can refer to reducing the expression of an RNA of the disease-causing gene, delivering a “normal” copy of a mutated gene to replace its expression, or modifying the mutant genome to “correct” a genetic defect^18^. All three of these genetic therapeutics have shown efficacy in experimental models, raising the possibility of successful gene therapy trials for fALS.

### Antisense oligonucleotides

Antisense oligonucleotides (ASOs) are short (13–25 nucleotides), synthetic, and single-stranded oligonucleotides that are designed to bind to specific sequences of RNA to reduce, restore, or modify RNA or protein expression^19^. The oligonucleotides typically contain chemical modifications that act to enhance pharmacokinetic properties and target binding affinity and the tolerability profile of the ASOs^20,21^. Depending on their target, binding sequence, and chemistry, single-stranded ASOs can modulate gene expression or modify pre-mRNA splicing through several distinct mechanisms of action, including target degradation, translational arrest, inhibition of RBP binding, splicing modulation, and altering translational activity^19,21^. Although ASOs do not cross the blood–brain barrier, they are effectively distributed throughout the central nervous system (CNS) when delivered into cerebrospinal fluid and have demonstrated promising results in the treatment of other neurological diseases^22–24^.

The first demonstration of a therapeutic potential in ALS was a 20-nucleotide ASO-targeting *SOD1*. Intra-cerebroventricular injections of this ASO in mutant *SOD1* rats resulted in the reduction of SOD1 mRNA and protein levels throughout the brain and spinal cord and slowed disease progression^25^. This ASO strategy of targeting SOD1 has since been tested in patients with SOD1 ALS in clinical trials. Intrathecal administration of the ASO (BIIB067, tofersen) was found to be safe and well tolerated and caused a significant reduction in SOD1 protein levels in the CNS in addition to slowing clinical decline in patients with SOD1 ALS^26^. Following these promising results, the ASO is being tested in patients with SOD1 ALS in a multicenter phase III placebo-controlled trial (NCT02623699).

For *C9orf72*-linked FTD/ALS, several ASO molecules have been designed and their beneficial effects have been demonstrated in numerous *C9orf72* cellular and animal models, including *C9orf72* patient induced pluripotent stem cell (iPSC)-derived neurons and *C9orf72* BAC transgenic mouse models^27–34^. Following the success of *C9orf72*-targeting ASOs in pre-clinical models, a phase I clinical trial of the first ASO (BIIB078) targeting *C9orf72* mRNA in *C9orf72* ALS patients was initiated in September 2018 and is ongoing (NCT03626012).

In addition to directly targeting ALS-causing mutations, pre-clinical experiments have tested ASOs targeting mRNAs that encode disease-modifying proteins, such as ataxin-2. Intermediate numbers (22–33 repeats) of the poly-glutamine repeat in *ATXN2* are associated with an increased risk of ALS^35,36^. Genetic knock-out or heterozygous deletion of *ATXN2* homologs can rescue TDP-43 toxicity in yeast, *Drosophila*, and a TDP-43 transgenic mouse, and a single administration of an ASO targeting ataxin-2 into the CNS of TDP-43 transgenic mice resulted in improved motor function and survival^35,37^. Given that accumulations of TDP-43 are by far the most common pathology seen in patients with ALS, these studies indicate that an ataxin-2-targeting ASO, if effective, has the potential to be therapeutically beneficial to large cohorts of patients with ALS, including patients with sALS.

### Adeno-associated viral vectors

The use of viral vectors to deliver genetic material required for gene replacement or knock-down is another therapy being explored in the treatment of ALS. Currently, the most frequently used viral vectors for neurodegenerative and other diseases of the CNS are adeno-associated viral (AAV) vectors^38^. AAV recombinant vectors are non-enveloped, single-stranded DNA-containing viruses that are modified such that the viral genome contains the desired therapeutic gene to be delivered along with only the necessary endogenous viral genes required for packaging^38^. AAV9 is the principal serotype used for the development of therapeutics in neurological diseases, including ALS, because of its high transduction efficiency in neurons, ability to spread broadly throughout the CNS, and its ability to cross the blood–brain barrier, enabling intravenous delivery^39^. AAV vectors can be used to deliver a “normal” copy of a disease-causing gene or to deliver RNA interference (RNAi) molecules—including small interfering RNAs (siRNAs), short hairpin RNAs (shRNAs), and microRNAs (miRNAs)—to downregulate and degrade mRNA transcripts of the targeted gene^38^.

Thus far, most ALS AAV vector therapies tested pre-clinically target mutant *SOD1*. Several groups have demonstrated the effective therapeutic potential of using AAV vectors to deliver miRNA or shRNA targeting mutant *SOD1* in SOD1^G93A^ rat and mouse models^40–44^. These studies all successfully demonstrated that AAV vector treatment resulted in knock-down of mutant SOD1 mRNA or protein expression (or both), slowed disease progression, and extended life span; one more recent study reported an increased median survival of 50%^40^. The safety and efficacy of intrathecal delivery of AAV vectors targeting mutant SOD1 have also been demonstrated in non-human primate models^41,44^ and more recently in two familial ALS patients carrying *SOD1* gene mutations^45^. Following these successes, an AAV9 viral vector containing an shRNA targeting mutant *SOD1* (AVXS-301) is under pre-clinical development and is expected to be tested in patients with SOD1 ALS in a phase I clinical trial in the near future^14^. Although AAV gene therapy is moving forward for ALS caused by *SOD1* mutations, it is important to note that *SOD1* mutations account for a small percentage of ALS cases, and it remains to be seen whether this therapy would also be beneficial to patients with sALS, as has been previously proposed^46^.

Targeting the more common genetic cause of ALS, *C9orf72*, with an AAV vector would be of benefit to a larger cohort of patients with ALS. The principle of using AAV9-miRNA to silence the *C9orf72* transcript was successfully demonstrated in cultures of primary cortical neurons derived from *C9orf72* BAC transgenic mice^47^. In addition, a more recent study has shown that an AAV5 vector containing miRNAs targeting repeat-containing *C9orf72* transcripts was able to reduce the accumulation of repeat-containing *C9orf72* transcripts in both the nucleus and cytoplasm of iPSC-derived frontal brain-like neurons^48^. Furthermore, intra-striatal delivery of these AAV5-miRNAs into 90-day-old *C9orf72* BAC transgenic mice lowered the expression of total and repeat-containing *C9orf72* mRNA transcripts, although no behavioral changes were observed in the mice following treatment^48^. Although reducing the levels of *C9orf72* transcripts has shown some promise in these experimental models, some concerns have been raised regarding the potential adverse effects caused by a reduction in the endogenous levels of the C9orf72 protein, and a recent study demonstrated that reducing C9orf72 function in a mouse model exacerbated phenotypes induced by the presence of the *C9orf72* repeat expansion^49^.

### CRISPR-Cas9 genome editing

CRISPR-Cas9 genome editing technology has rapidly advanced within the last decade, making it a potential therapeutic option for human diseases arising from genetic mutations. This technology makes use of a naturally occurring prokaryotic defense mechanism to insert, modify, or remove specific sequences of DNA using a targeting guide RNA, a Cas9 DNA endonuclease, and the cells’ natural DNA repair mechanisms^50^. In a therapeutic setting, these components would most likely be delivered via an AAV vector, adopting the principles learnt from AAV-mediated gene therapies^51^.

For ALS cases caused by the *C9orf72* mutation, the most likely CRISPR-Cas9-mediated approach would be to remove the hexanucleotide repeat expansion sequence. As proof of concept, this approach has been used successfully to generate isogenic *C9orf72* patient-derived iPSCs^52–54^. CRISPR-Cas9 excision of the repeat sequence reduces some of the pathological hallmarks of the diseases, including repeat RNA foci and DPR proteins. A recent study has also demonstrated that a similar reduction in DPR proteins and a rescue of neurodegeneration can be achieved by using CRISPR to selectively delete the *C9orf72* promoter region^55^. However, before CRISPR-Cas9 technology may be of therapeutic use in *C9orf72*-linked ALS, it will be important to address the problem of potentially introducing insertion/deletion events into the wild-type allele, which may have functional consequences to the *C9orf72* gene^56^. Finally, a recent study successfully targeted the Cas9 protein to the repeat RNA instead of the DNA in an attempt to circumvent permanent genomic changes^57^.

For SOD1 ALS, it may be possible to use CRISPR-Cas9 genome editing to correct specific disease-causing mutations, delete the mutant *SOD1* gene, or introduce strategic mutations to disable the mutant SOD1 function. This approach has been successful in the SOD1^G93A^ mouse model where CRISPR/Cas9 editing *in vivo* reduced expression of mutant SOD1, delayed disease onset, and increased survival^58,59^. AAV-delivered CRISPR-Cas9 technology has also been tested in combination with a cytidine base editor to introduce a nonsense coding mutation into the mutant *SOD1* gene to permanently disable SOD1 expression in the SOD1^G93A^ mouse model. This treatment slowed disease progression, prolonged survival, and caused 40% fewer SOD1 inclusions in end-stage mice compared with control^60^.

Further *in vitro* and *in vivo* validation work is required to establish the feasibility and tolerability CRISPR-Cas9-mediated therapeutics in fALS as a number of challenges associated with genome editing technology—such as target specificity, off-target genome editing, and immunogenicity—would need to be overcome before it could be a viable therapeutic. Additionally, there would need to be significant ethical and regulatory changes put in place before this technology enters the clinic.

## Modulating neuroinflammation

In addition to motor neuron death and muscle denervation, a characteristic feature of ALS pathology is neuroinflammation^61,62^. Increasing evidence from ALS patient tissue and animal studies has implicated activation of astrocytes, microglia, and the complement system, T-lymphocyte infiltration and production of inflammatory cytokines in ALS pathogenesis^61–64^. Furthermore, numerous *in vitro* studies of motor neurons co-cultured with astrocytes, microglia, or T cells derived from iPSCs carrying an ALS-causing mutation or ALS mouse models have demonstrated that these cells have a toxic effect on motor neurons^46,65–69^. Given the increased activation of the immune system in ALS, several clinical trials have investigated using various classes of anti-inflammatory drugs as an ALS therapeutic. Although many anti-inflammatory drugs have failed to demonstrate clinical efficacy, a number of trials remain ongoing and different aspects of the immune system are being targeted^61,70^.

Modulating aberrant activation of microglia is the target of many of the drugs currently in trials. Microglia are the resident immune cells of the CNS and are classically described to exist in two different states: resting and activated. Whereas resting microglia survey their microenvironment and perform crucial roles to maintain homeostasis, activated microglia react rapidly to environmental abnormalities and can be both protective and detrimental to the surrounding cellular environment, a phenomenon that is sometimes referred to as M1 or M2 phenotypes^71^. Microglia are considered to have a “toxic” phenotype when they are responsible for the release of pro-inflammatory cytokines and are now often referred to as disease-associated microglia^72,73^. At the same time, microglia show neuroprotective properties when they release anti-inflammatory cytokines and remove cellular debris by phagocytosis^72^. Aberrant activation and an imbalance of toxic and protective microglia states are thought to promote inflammation and motor neuron degeneration in ALS; thus, anti-inflammatory drugs that reduce microglia activation or induce a protective microglia phenotype are being explored as therapeutic options^61,71^.

Masitinib is a tyrosine kinase inhibitor that reduces microglia proliferation and activation. It has shown promising results in both pre-clinical and clinical trials. Oral administration of masitinib decreased microgliosis, reduced motor neuron pathology, and prolonged post-paralysis survival in SOD1^G93A^ mice^74^. When translated into a phase II/III trial (NCT02588677), masitinib in combination with riluzole demonstrated a slowed decline of the Revised ALS Functional Rating Scale (ALSFRS-R) in patients with “normal progressor” ALS^75^. A further phase III (NCT03127267) trial of masitinib in combination with riluzole is due to commence shortly.

Ibudilast has also recently gained approval to be tested in a phase IIb/III trial (NCT04057898) in patients with ALS after a phase II trial demonstrated that the drug was safe and had potential benefits on survival^76^. Ibudilast is a small-molecule inhibitor of phosphodiesterase 4 and 10 and Toll-like receptor 4 and is thought to promote an anti-inflammatory effect. Interestingly, ibudilast has also been shown to significantly enhance the clearance of TDP-43 and SOD1 protein aggregates and protect against TDP-43-mediated toxicity in cell culture models, suggesting that this drug may exhibit more than anti-inflammatory properties^77^.

Fasudil is another drug that acts to modulate microglia activation and phenotype. It is a Rho kinase (ROCK) inhibitor that reduces the release of pro-inflammatory cytokines and has been shown to promote expression of neuroprotective microglia markers upon stimulation in cellular models and significantly prolong survival and motor function by modulation of microglial activity in SOD1^G93A^ mice^78–80^. Fasudil is being investigated in a phase IIa clinical trial (NCT03792490) in patients with early-stage ALS^81^. A recent case study of three patients who were granted compassionate treatment of fasudil before the trial began has reported that the drug was well tolerated; however, no conclusions with regard to drug efficacy could be drawn^82^. This will be assessed via several secondary endpoint measures in the ongoing phase IIa trial^81^.

In addition to targeting microglia, anti-inflammatory drugs that target the complement system are being tested in patients with ALS. The complement system is part of the innate immune system which acts to enhance the immune response. The system consists of several small proteins that circulate in the blood as inactive precursors which become activated by proteases upon stimulation by one of several triggers^83^. In ALS, there is increasing evidence of aberrant activation of various components of the complement system in the onset and progression of motor phenotypes^83,84^. The terminal protein of the complement system, complement component 5 (C5), has been identified as a potential therapeutic target in ALS on the basis of evidence of C5a receptor upregulation in post-mortem ALS tissue and SOD1^G93A^ animal models and the fact that pharmacological inhibition of the C5a receptor improved symptoms and prolonged survival in SOD1^G93A^ mice^85,86^. Clinical trials in patients with ALS are planned for two drugs acting on C5. A phase III trial (NCT04248465) is under way in both sALS and fALS patients to test the efficacy and safety of ravulizumab, a humanized monoclonal antibody designed to bind to and inhibit the activation of C5. Similarly, a phase II trial (NCT04297683) in both sALS and fALS patients is planned for a synthetic peptide inhibitor of C5 activation, zilucoplan.

A third intervention targeting neuroinflammation in ALS is suppression of the effects of pro-inflammatory cytokines by antagonizing cytokine receptors. This is an effective strategy already used in the treatment of inflammatory diseases such as rheumatoid arthritis, and several of these anti-inflammatory drugs are being trialed for repurpose in ALS. Examples include the interleukin-1 (IL-1) receptor antagonist, anakinra (NCT01277315), and tocilizumab (NCT02469896), a monoclonal antibody targeting the IL-6 receptor. Pilot studies in a small number of patients with sALS indicate that anakinra and tocilizumab can lower cytokine levels and induce a down-regulation of inflammatory genes, respectively^87,88^.

## Immunotherapy

Protein aggregates are a pathological hallmark of all cases of ALS regardless of disease etiology and include, but are not exclusive to, SOD1, FUS, TDP-43, but also C9orf72 repeat translated DPR proteins. In experimental models of several other neurodegenerative disorders, antibody-based therapies have been effective in reducing the cell-to-cell transmission of toxic proteins—such as tau, amyloid-β, and α-synuclein—and removal of pathological aggregates of these proteins^89,90^. A similar immunotherapy approach has been explored to reduce protein aggregation in models of ALS. This either involves direct injection of a purified antibody into the target organism (passive immunity) or can involve a vaccination approach whereby the target organism is injected with a recombinant form of the toxic protein in order to stimulate *in vivo* antibody production against the target protein (active immunity).

For SOD1 ALS, several studies have demonstrated the benefits of both active and passive immunotherapy in mutant SOD1 models, although the success of injecting recombinant SOD1 mutant protein to induce immunity seems to be dependent on the *SOD1* mutation. Although vaccination against SOD1 has been shown to delay disease onset, increase life span, and enhance the clearance of SOD1 aggregates in the SOD1^G37R^ model^91–93^, this immunization strategy has not been successful in extending life span in the SOD1^G93A^ mouse model, and some studies have reported a worsening of disease phenotype, likely due to adverse immune responses^91,94,95^. In contrast, studies that used a passive immunity approach by treating mutant SOD1 mouse models with various types of antibodies specific for misfolded SOD1 all reported delayed disease onset, increased life span, and a reduction in mutant SOD1 protein levels^96–98^.

In *C9orf72*-linked ALS, antibodies targeting DPR proteins, in particular poly-glycine-alanine (GA), have shown promising effects in experimental models. Antibodies raised against the poly-GA protein are able to reduce intracellular poly-GA aggregation in primary neuron cultures and blocked the seeding activity of brain lysates extracted from *C9orf72* patient brain^99^. Recent studies have further demonstrated beneficial effects of anti-GA antibodies in two *C9orf72* mouse models, as shown by reduced behavioral deficits, decreased neuroinflammation, and prolonged survival^100^, as well as reduced TDP-43 cytoplasmic mislocalization, suggesting that the immunotherapy was able to elicit effects downstream of poly-GA aggregation^101^. These studies support the concept that poly-GA immunotherapy could be a viable therapeutic approach in reducing some aspects of *C9orf72*-linked ALS; however, given the multi-factorial disease pathogenesis of *C9orf72*, it is likely that treatment will require a synergistic approach when targeting downstream mechanisms.

Given that most ALS cases present with TDP-43 pathology, antibodies targeting this protein would be of therapeutic benefit to a larger population of patients with ALS, including sALS. Indeed, single-chain antibodies that recognize different regions of TDP-43 have been investigated as potential therapeutics^102,103^. In mutant TDP-43 transfected cell lines, an antibody targeting the nuclear export signal of TDP-43 was found to have high affinity for and accelerated proteasome-mediated degradation of aggregated TDP-43. Furthermore, following *in utero* electroporation in embryonic mouse brain, the antibody caused a marked reduction in the number and size of mutant TDP-43 aggregates^102^. Similarly, an antibody targeting the RNA recognition motif of TDP-43 significantly reduced TDP-43 proteinopathy, motor defects, and neuroinflammation in transgenic mice expressing ALS-linked TDP-43 mutations^103^. It is important to note that mutant TDP-43 was studied in both cases. Given that mutations in TDP-43 account for only 5% of fALS cases, it will be important to assess whether antibody-based therapy is able to reduce aggregation of the wild-type protein, as this is the more common TDP-43 protein species found in ALS patients with TDP-43 proteinopathy.

## Stimulating clearance of protein aggregates

An alternative therapeutic strategy aimed at the disassembly of protein aggregates is based on enhancing protein quality control systems via heat shock proteins. These proteins are essential to intracellular protein quality control and function as molecular chaperones to correctly fold, stabilize, and prevent the unwanted aggregation of proteins^104^. Arimoclomol, a compound that induces the upregulation of several heat shock proteins, has shown therapeutic benefit in the SOD1^G93A^ mouse model of ALS^105^. Administration of arimoclomol significantly improved muscle strength, motor neuron survival, and prolonged life span compared with controls when treated from the time of symptom onset^106,107^. Histopathological analysis of arimoclomol-treated mice revealed a reduction in the abundance of ubiquitin-positive aggregates in motor neurons compared with untreated mice, suggesting that arimoclomol was acting as an anti-aggregation drug in this model of ALS^107^. In patients with rapidly progressing SOD1 ALS, arimoclomol was found to be safe and well tolerated and provided therapeutic benefit across a range of efficacy outcome measures in a randomized, double-blind, placebo-controlled trial^108^. A larger randomized phase 3 clinical trial to evaluate the efficacy and safety of arimoclomol in patients with sporadic and familial ALS is under way (NCT03491462). Whilst arimoclomol has demonstrated therapeutic benefit in SOD1 ALS, it will be particularly interesting to see the effect of arimoclomol on non-SOD1 ALS patients given that pre-clinical research on arimoclomol in non-SOD1 ALS is currently limited.

One molecular chaperone that is of particular interest with regard to ALS therapeutics is HSPB8. This protein has been shown to recognize and promote the autophagy-mediated removal of misfolded TDP-43 fragments, mutant SOD1, and C9orf72 DPR proteins^109–111^. Furthermore, HSPB8 can form a chaperone complex with BAG3 and HSP70, the latter of which acts as a key regulator of stress granule surveillance to help prevent the conversion of dynamic stress granules into more solid aggregates^112^. HSPB8 in increasing levels is being explored as an ALS therapeutic in a phase II clinical trial of colchicine (NCT03693781), which is known to enhance the expression of HSPB8 and several other proteins involved in autophagy as well as exhibiting anti-inflammatory properties^113^.

An additional molecular chaperone of therapeutic interest is Hsp104, a heat shock protein with disaggregase activity that is naturally found in *Saccharomyces cerevisiae*, where it functions to regulate the construction and disassembly of yeast prion proteins^114,115^. Interestingly, despite Hsp104 being conserved between bacteria and many eukaryotes, no homolog of Hsp104 exists within the animal kingdom, making it an attractive candidate as an exogenous therapeutic^116,117^. Although wild-type Hsp104 has only moderate effects on the disaggregation of human proteins associated with neurodegenerative diseases, variants of Hsp104 have been engineered to suppress TDP-43 and FUS aggregation and toxicity in yeast models^118–120^. Furthermore, co-expression of Hsp104 variants with ALS-linked mutant FUS in mammalian cells promoted the dissolution of FUS inclusions^121^. Further investigation is required to determine whether Hsp104 variants have the same effect on TDP-43 aggregates in mammalian cells and whether this extends to *in vivo* ALS models. It will also be necessary to determine whether expression of Hsp104 in mammalian cells has any detrimental effects on the normal protein folding process before this can be pursued as a therapeutic^15^.

A less targeted approach to stimulating the disassembly of pathological protein aggregates would be to increase the activity of specific cellular protein quality control pathways. Several genes linked to ALS are known to play a role in autophagy, which has led to the suggestion that dysfunctional autophagy may influence disease pathogenesis and thus therapeutic strategies aimed at enhancing autophagy could be an effective treatment for the disease. Several studies have demonstrated that stimulating autophagy can enhance the clearance of mutant SOD1, TDP-43, or FUS *in vitro*; however, results in *in vivo* models have been mixed^122–128^. Despite these conflicting pre-clinical studies, a placebo-controlled phase 2 clinical trial to assess the biological and clinical effect of the potent autophagy-inducing drug rapamycin is ongoing in patients with ALS (NCT03359538).

## Targeting phase separation

In addition to stimulating the disassembly of protein aggregates, another therapeutic strategy could be to prevent the initial formation of these structures by interfering with the process of liquid–liquid phase separation (LLPS). This is a naturally occurring phenomenon that underlies the formation of various membrane-less organelles, such as the nucleolus and stress granules, and occurs when proteins demix from an aqueous solution and form dynamic liquid-like droplets. This process is believed to be mediated by low-complexity domains within the phase-separating protein, a property that is common among many of the RBPs implicated in ALS, including TDP-43, FUS, and hnRNPA1^129–131^. While LLPS is essential to maintain cellular function, recent *in vitro* and cellular studies have demonstrated that liquid-like droplets are capable of solidifying over time, resulting in the formation of insoluble aggregates, which can have toxic consequences for the cell^132,133^. Given that many of aggregated proteins detected in patients with ALS are capable of this phenomenon, a therapeutic strategy being explored in pre-clinical experiments is interfering with LLPS to prevent the deleterious process of liquid-like droplets, particularly stress granules, from forming insoluble aggregates. Although no clinical translation of these studies is planned at present, these pre-clinical experiments demonstrate how an understanding of the basic biology and biochemistry of the disease can lead to the emergence of novel therapeutic strategies.

The use of RNA bait oligonucleotides is one strategy being explored after a study demonstrated that binding of RNA to TDP-43 suppresses LLPS and inclusion formation in a light-inducible model of TDP-43 LLPS^134^. Treatment of cortical-like neuronal cells with a bait RNA oligonucleotide that exhibited high affinity for the RNA recognition motif of TDP-43 suppressed phase transition of TDP-43 and reduced neurotoxicity in a dose-dependent manner. The use of such RNA oligonucleotides could be a viable therapeutic treatment as they could be readily administered to patients in a similar manner to the ASO therapies currently being trialed. Another therapeutic option targeting phase separation would be using small molecules. A number of studies have identified compounds that show anti-TDP-43 aggregation properties^135–137^. A recent small-molecule screen identified several planar compounds capable of modulating stress granule size, number, and dynamics and preventing the accumulation of TDP-43 within persistent cytoplasmic puncta in mutant TDP-43 iPSC-derived motor neurons^138^. Although both strategies are effective in reducing the deleterious accumulation of TDP-43, caution must be taken with this strategy not to interfere with the normal physiological function of phase separation and formation of essential membrane-less organelles.

Targeting post-translational modifications (PTMs) of phase-separating proteins may also be a therapeutic strategy as some modifications have been found to influence the aggregation propensity of ALS-linked proteins. Poly(ADP-ribosyl)ation is an important PTM in the regulation of stress granule dynamics, and it was recently discovered that TDP-43 and hnRNPA1 are targets of poly(ADP-ribose) polymerases (PARPs)^139,140^. The addition of poly(ADP-ribose) units to these proteins enhances their phase separation, while PARP inhibition is able to reduce accumulation of cytoplasmic TDP-43 foci and rescue TDP-43-associated neurotoxicity in NSC-34 cells, rat primary spinal cord neurons, and *Drosophila* models of ALS^139–141^. These studies suggest that inhibition of PARPs could be an effective strategy to regulate aberrant phase separation in ALS, and given that PARP inhibitors are already safely used clinically for the treatment of various cancers, this could be a viable therapeutic option^142^. Arginine methylation is another PTM known to regulate the phase separation of the ALS-linked protein FUS. The addition of methyl groups to arginine residues within FUS by protein arginine methyltransferase (PRMT) enzymes reduces the ability of FUS to phase-separate and form hydrogels *in vitro*^143,144^. Treatment with select concentrations of a global methyltransferase inhibitor has been shown to mitigate the cytoplasmic mislocalization and aggregation of mutant FUS in a cell culture system^145^. However, caution is warranted with increasing PRMT activity as these enzymes target a large number of essential proteins that would likely be affected by this treatment. However, the discovery that PTMs can have a profound effect on the behavior of aggregation-prone proteins opens up the possibility of therapeutically manipulating a range of different enzymes to reduce the aberrant phase separation of aggregation-prone proteins. Thus, PTMs of ALS-associated proteins should be further explored to uncover potential therapeutic targets.

## Targeting nucleocytoplasmic transport

Deficits in nucleocytoplasmic transport have been widely reported in several models of ALS as well as other neurodegenerative disorders^146^. The most common defect seen in ALS patients with TDP-43 pathology is the depletion of TDP-43 from the nucleus, resulting in loss of essential nuclear functions of the RBP. In a healthy neuron, TDP-43 can rapidly traffic between the nucleus and cytoplasm; however, pathological depletion of TDP-43 from the nucleus is toxic to neurons, both *in vitro* and *in vivo*^147,148^. A similar phenomenon is observed in patients with ALS-FUS, where the predominantly nuclear FUS protein is mislocalized from the nucleus to the cytoplasm^149^. Thus, preventing the nuclear export or enhancing nuclear import of these proteins has been investigated as a therapeutic strategy for ALS.

TDP-43 contains a canonical nuclear export signal (NES) that is predicted to be recognized by the nuclear export factor exportin 1 (XPO1/CRM1), a nuclear export pathway that can be inhibited by selective inhibitors of nuclear export (SINE) compounds. Inhibition of XPO1 by SINE compounds has been shown to reduce TDP-43-induced cortical neuron death, rescue larval locomotor defects, and partially restore motor function in a TDP-43 overexpressing rat model^150,151^. Additionally, genetic knockdown of XPO1 in a *Drosophila* can rescue disease phenotypes associated with FUS or G_4_C_2_ repeat-induced neurotoxicity^33,152^. Interestingly, treatment with SINE compounds or siRNA depletion of nuclear export proteins had limited effects on the nuclear localization of TDP-43 in rat primary cortical neurons at concentrations shown to rescue TDP-43-induced toxicity^150^. This observation is in line with recent studies that have suggested that the NES of TDP-43 is not functional and most TDP-43 export from the nucleus is likely a result of passive diffusion^153,154^. Therefore, it appears that the protective effects of SINEs may be mediated through other unknown mechanisms that are independent of TDP-43 localization. Despite currently lacking a clear mechanism of action, a small-molecule inhibitor of XPO1, BIIB100/KPT-350, is being tested in a placebo-controlled phase 1 clinical trial in patients with ALS (NCT03945279).

Recently, it has been shown that select nuclear import receptors are capable of antagonizing aberrant phase separation and stimulating the disassembly of protein aggregates. Several groups have demonstrated that, in addition to its nuclear import function, karyopherin β2/transportin 1 functions as a molecular chaperone to prevent FUS aggregation by interfering with its ability to phase-separate and can disassemble preformed fibrils and hydrogels of FUS *in vitro*^143,144,155–157^. Overexpression of karyopherin β2 also reduced toxicity in both human cells expressing mutant FUS and mutant FUS *Drosophila* models^155^. Additionally, karyopherin β2 has been shown to inhibit and reverse the fibrillization of several other RBPs linked to ALS/FTD, including TAF15, EWSR1, hnRNPA1, and hnRNAP2^155^. Although this strategy is far from any clinical use, the utilization of nuclear importers as an antagonist for aberrant phase separation demonstrates the innovative potential of therapeutic strategies that have emerged from recent advances in our understanding of ALS biology.

## Emergence of new clinical trial design for ALS therapies

The search for effective ALS therapies is not only dependent on advances in understanding disease biology and the emergence of novel targets, it is also reliant on well-designed and executed clinical trials. The majority of ALS clinical trials to date have followed the often costly and time-consuming, traditional randomized clinical trial design of testing a single treatment in a homogenous group of patients. As it has become clear that ALS is a multi-faceted and heterogeneous disease, at both the clinical and molecular levels, a need for a new trial design is warranted. Trials for other diseases, particularly cancers, have benefitted from the use of a “platform trial” design^158–161^. A platform trial is a clinical trial with a single master protocol in which multiple treatments are evaluated simultaneously across one or more types of patients and allows for further additions or exclusions of new therapies or patient populations during the trial^158,162^. This model accelerates the development of therapies by rapidly evaluating the effectiveness of multiple therapies simultaneously, increasing patient access, and lowering the cost of trials. Furthermore, data from all patients receiving a placebo in each group can be combined, enhancing the statistical power of the trial. In January 2020, the US Food and Drug Administration granted approval for the first platform trial in patients with ALS. Initially, the HEALEY ALS platform trial (NCT04297683) will involve simultaneously trialing three potential therapeutic drugs, and new treatments and additional participants will be added as they become available. This trial design is estimated to decrease the trial time by 50%, increase patient participation by 67%, and reduce the cost of research by 30%^163^. It is hoped that use of the platform trial model will help accelerate the development of effective ALS therapies.

## Summary

It is clear from the number of identified therapeutic targets discussed here that ALS is a complex and heterogeneous disease for which the development of therapies has been a challenging process. Nevertheless, significant progress made within the last few years has increased understanding of the genetics, pathology, and mechanisms of disease and led to the emergence of a range of novel therapeutic strategies, many of which are now entering clinical trials. As ALS research advances, it is becoming clear that the different forms of ALS exhibit unique molecular characteristics and pathologies—for example, DPR proteins in *C9orf72*-ALS and FUS aggregates in *FUS*-ALS—yet they all share common motor neuron neurodegeneration and inflammatory features that make them clinically indistinguishable. These similarities and differences have given rise to the development of therapeutic strategies that are highly specific, targeted to a discrete pathological mechanism, as well as strategies that affect features common to all patients with ALS. As understanding of ALS pathobiology increases, it is becoming more unlikely that a single therapeutic will prove to be effective in treating all forms of ALS caused by different underlying molecular differences. Instead, a more appropriate approach may be to use a combination of synergistic therapeutics to target different aspects of disease pathogenesis. However, therapies that target common features of the disease, such as inflammation and protein aggregate clearance, could prove to be useful adjunct therapies that modify disease progression. To complement this approach, the introduction of a platform trial design to ALS trials will hopefully accelerate the search to find effective therapies as it allows fast assessment of multiple therapeutic strategies. As many of the therapies discussed here enter or continue through clinical trials within the next few years, it will become clearer which therapeutic strategies, or potential combinations, are likely to make the most significant impact on disease outcomes.
